# Influence of Needle Design and Irrigant Flow Rate on the Removal of *Enterococcus faecalis* Biofilms In Vitro

**DOI:** 10.3390/dj10040059

**Published:** 2022-04-02

**Authors:** Charley Provoost, Giovanni Tommaso Rocca, Anna Thibault, Pierre Machtou, Serge Bouilllaguet

**Affiliations:** University Clinics of Dental Medicine, University of Geneva, 1207 Geneva, Switzerland; charleyprovoost@gmail.com (C.P.); giovanni.rocca@unige.ch (G.T.R.); anna.thibault@unige.ch (A.T.); prpierre.machtou@gmail.com (P.M.)

**Keywords:** *E. faecalis* biofilms, endodontic irrigation, needle

## Abstract

This study aimed to evaluate the influence of needle design and irrigant flow rate on the removal of *Enterococcus faecalis* mature biofilms during sodium hypochlorite irrigation. Forty-eight single-rooted human teeth were instrumented (ProTaper F3), autoclaved and inoculated with *Enterococcus faecalis* to establish a two-week-old biofilm. *E. faecalis* biofilms were treated with Sodium hypochlorite that was injected in the root canals using three types of needles (NaviTip, ProRinse, IrriFlex). For the IrriFlex needle, one, two, or four bars of pressure was applied to the irrigating solution to increase flow rates. Bacteria were labeled with the LIVE/DEAD BacLight Bacterial Viability kit, and viability was assessed by flow cytometry (FCM). Results were statistically analyzed using one-way ANOVA and Tukey multiple comparison intervals (α = 0.05). Bacterial viability was significantly reduced after sodium hypochlorite passive irrigation but the number of viable bacteria retrieved from root canal specimens irrigated with the Pro-Rinse needle was significantly higher compared to NaviTip and IrriFlex needles (*p* < 0.05). When the irrigant flow rate was increased, the viability of bacterial biofilms was significantly reduced compared to passive irrigation using the IrriFlex needle (*p* < 0.05). Applying higher flow rates during irrigation using the IrriFlex needle did not further reduce bacterial viability.

## 1. Introduction

Following pulpal infection and necrosis, bacteria colonize the root canal space, adhere to root canal dentin and encapsule themselves in a self-produced matrix of extracellular polymeric substances to form a biofilm [1]. Growth inside a biofilm helps bacteria to survive the antibacterial action of sodium hypochlorite which is used during root canal irrigation. Whereas sodium hypochlorite rapidly oxidizes membranes of bacteria located on top of the biofilm, deeply embedded bacteria are more likely to survive because they remain protected from the direct action of chlorine. Bacterial biofilms also better resist the flushing action of irrigating solutions which are injected into the canal space. As reported by Gulabilava et al. [2], endodontic irrigants should exert shear stress on canal walls to detach the adherent biofilm and to remove the smear layer. Other reports showing that higher hydrodynamic shear stresses more efficiently remove root canal biofilms and contaminants further support the concept of a chemo-mechanical action of irrigating solutions.

Clinically, a needle-and-syringe system is used to inject the solution into the depth of the root canal. Current literature suggests that the size and the design of the needle, the insertion depth of the needle tip and the flow rate of the solution are all factors affecting the cleansing of the root canal and the elimination of bacterial biofilms [3,4]. Although endodontic irrigating solutions are intended to be contained within the root canal, they are sometimes extruded through the apical foramina during irrigation. When narrow 30-G open-ended needles are deeply inserted in the root canal, they generate a high flow rate of irrigant directed toward the apex of the tooth, which may increase the risk of periapical extrusion [5]. Apical extrusion of irrigating solutions can be a serious complication during root canal therapy [6].

In response to this potential risk of extrusion, side-vented needles that deliver the solution laterally have been developed. However, their efficacy at removing bacterial biofilms has been criticized because their action was shown to be limited to the area adjacent to the side opening of the needle [7]. Also, they generate lower flow rates than conventional open-ended needles placed at the same depth inside the root canal, as shown using a computational fluid dynamics model.

Most recently, a polyethylene flexible needle exhibiting two back-to back side vents and a closed-end has been introduced into the market. This double-sided vented needle allows the production of two jets oriented in an oblique direction, and delivers a large volume of irrigating solution at a high flow rate with less risk of apical extrusion. However, the efficiency of this new plastic needle remains to be studied.

*Enterococcus faecalis* is frequently retrieved from infected root canals, sustains endodontic infection and contributes to failure after treatment [8]. *E. faecalis* has the ability to deeply penetrate inside dentin tubules and to form biofilms along root canal walls [9,10]. Its extracellular polymeric matrix plays a crucial role in allowing the biofilm to resist external forces applied by the irrigant flow generated during irrigation. Because of its contribution to endodontic infection and its adaptive capabilities to severe environmental conditions, *E. faecalis* mature biofilms are frequently used as a model of endodontic infection for ex vivo studies [11].

This study aimed to evaluate the influence of needle design and irrigant flow rate on the removal of *Enterococcus faecalis* mature biofilms subjected to sodium hypochlorite irrigation. The hypotheses tested were that the elimination of intracanal *E. faecalis* biofilms (1) would be improved when using a new double-sided vented plastic irrigation needle, and (2) would be increased when higher irrigant flow rates are generated using the double-sided vented plastic irrigation needle.

## 2. Materials and Methods

### 2.1. Specimen Preparation

Single rooted-teeth used in this study were collected at the Oral Surgery Department of the University of Geneva and immediately frozen after extraction. According to the local regulations, ethics approval has been obtained for the use of anonymous extracted teeth for research purposes. The Ethics committee authorizes the use of biological tissues which have no link with patient identity.

**Figure 1:**
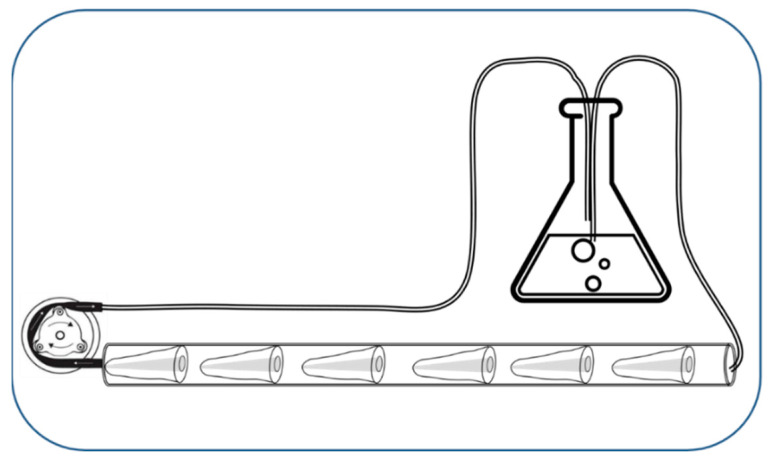
Model used to establish a two-week-old bacterial biofilm. The culture medium was circulated using the peristaltic pump and renewed at different time intervals to remove dead bacteria and debris. Each root sample was used separately for the irrigation procedures.

The teeth had a similar apical foramen diameter as verified under the operating microscope (Zeiss Extaro 300, Oberkochen, Germany), and no previous root filling. Teeth were decoronated with a water-cooled diamond disc (Kerr, Bioggio, Switzerland) to obtain 16 mm long sections of root. The working length of each root was established using a size 10 K-file that was placed 1 mm short of the apical foramen. Root canals were mechanically enlarged using ProTaper Gold (Dentsply-Sirona Endodontics, Ballaigues, Switzerland) under 1% NaOCl irrigation. Between each instrument, apical patency was verified by passing a size 10 K-file through the foramen; the final preparation was made using ProTaper Gold F3. After preparation was complete, 17% EDTA was applied for 1 min to dissolve the smear layer, then quickly neutralized with NaOCl and rinsed with distilled water (5 mL) [12,13,14]. The samples were then sonicated 2 × 15 min in double-distilled sterile water and tightly fitted into a thick-walled silicone tube having a 4 mm internal diameter (Semadeni, Ostermundigen, Switzerland). A set of six roots was placed at equal distance from each other into a 20 cm long piece of silicon tube (Figure 1), that was filled with double distilled water and autoclaved at 121 °C for 40 min (Tomy SX-500E, Tokyo, Japan).

### 2.2. Root Canal Infection with E. faecalis

*E. faecalis* (HUG 135737) was grown overnight on Columbia agar plates, then suspended in BHI broth (BHI, Becton Dickinson, Allschwil, Switzerland) and allowed to grow for 4 h at 37 °C. The cell density of the suspension was spectrophotometrically adjusted to an optical density of 0.2 at 600 nm. The bacterial suspension was introduced into the sterilized silicon tube containing six root samples and placed into the incubator at 37 °C (HeraCell 150, Thermo Fisher Scientific, Inc., Reinach, Switzerland). The peristaltic pump was activated to completely fill the circuit and to eliminate entrapped air bubbles. The bacterial suspension was recirculated twice a day, at a minimum flow rate of 100 μL/min. After 48 h, the bacterial suspension was removed, and the circuit was filled with fresh BHI medium that was recirculated two times per day over one week (Figure 1). Thereafter, the circuit was filled with phosphate-buffered saline (PBS, Gibco, Thermo Fisher Scientific Inc., Reinach, Switzerland) that was was recirculated for 2 min, twice a day, until the end of the second week. This nutrient depleted phase was adopted to promote the maturation of biofilms because mature biofilms better resist antibacterial treatments [15].

On the day of the experiment, the hydraulic circuit was rinsed with fresh PBS and disconnected from the peristaltic pump. Each root was separated from the other by sectioning the silicone tubing 5 mm above the coronal entrance of the root to maintain a fluid reservoir over the root canal for further irrigation. At the apical level, the external root surface was wiped with sterile gauze that was soaked into sodium hypochlorite. The apical foramen was then embedded into a light-body silicon material to facilitate handling of the specimens during irrigation and to reproduce a closed-end system [16].

### 2.3. Root Canal Irrigation

Root canal irrigation was performed using a 10 mL syringe (Plastipak, Franklin Lakes, NJ, USA) connected to three different irrigating needles, all having an external diameter of 0.3 mm (30 G). The NaviTip needle (Open-ended, Ultradent, South Jordan, UT, USA) and the ProRinse needle (Side-vented, Dentsply-Maillefer, Ballaigues, Switzerland) were used for passive irrigation. The experimental needle (IrriFlex, Produits Dentaires SA, Vevey, Switzerland), a double-side-vented polypropylene needle, was used for passive irrigation and for delivering sodium hypochlorite at various flow rates. This was made possible by connecting the syringe/IrriFlex needle to an automatic dispensing unit that delivered the liquid at various pressures and dispensing times (D3PDSD-02, Sigrist & Partner AG, Matzingen, Switzerland). Three pressure conditions (1, 2, 4 bar) were selected to deliver sodium hypochlorite.

During passive irrigation, the needle tip was inserted at 1 mm from the apical foramen and moved back and forth until 2 mL of 1% sodium hypochlorite was delivered in 30 s (flow rate: 0.06 mL s^−1^). When the IrriFlex needle was connected to the pressure apparatus, the irrigation time was adjusted at 15 s for 1 bar pressure (flow rate: 0.13 mL s^−1^), 10 s for 2 bar (flow rate: 0.2 mL s^−1^), and 6 sec for 4 bar (0.33 mL s^−1^). Thus, the total volume of sodium hypochlorite introduced into root canals was kept constant among groups; only flow rates were modified. All irrigation procedures were performed under a laminar hood to avoid external contamination.

After the irrigation process was complete, root canals were rinsed with PBS, which was completely suctioned off before adding 100 µL of fresh solution. A sterile #15 K file was placed at the working length and used to gently scrape canal walls. Then, a sterile paper point was used to absorb the solution. Both the K file and the paper point were transferred into a sterile tube containing 1 mL of NaCl that was sonicated for 20 s to disperse bacterial aggregates and to generate single-cell suspensions [17].

Immediately after ultrasonic treatment, bacterial viability was measured using the LIVE/DEAD BacLight Kit (Life technologies Europe BV, Zug, Switzerland) comprising two fluorescent nucleic acid stains, SYTO-9 (green) and propidium iodide (red) indicative of cell membrane integrity. When propidium iodide enters a damaged cell, it displaces the SYTO 9 from the bacterial DNA, therefore shifting the fluorescence from green to red [18]. Two hundred microliters of SYTO 9 and PI stain were mixed with the same volume of bacterial suspension and incubated at room temperature in the dark for 15 min. The flow cytometry analysis was performed using a BD Accuri C6 flow cytometer (BD Accuri cytometers, Ann Arbor, MI, USA) having a 488 nm wavelength laser for excitation of both dyes. Fluorescence emission of SYTO 9 has been collected in the FL1 channel (BP 533/30) and PI in the FL3 channel (LP > 670). Bacterial density and flow rate (14 mL/min) were set to keep the event rate below 800 events/sec during acquisition. The data were analyzed with FlowJo software (FlowJo for Windows, version 10.0.06, 2014, Tree Star Inc., Ashland, OR, USA) and results were expressed as a percentage of viable cells among the total number of cells collected in the suspension. Results were statistically analyzed (IBM SPSS Statistics 27.0.1.0, Academic authorized user (5725-A54)) using one-way ANOVA and Tukey multiple comparison intervals (α = 0.05). Each experiment was conducted in triplicate and repeated five times (*n* = 15).

## 3. Results

**Figure 2:**
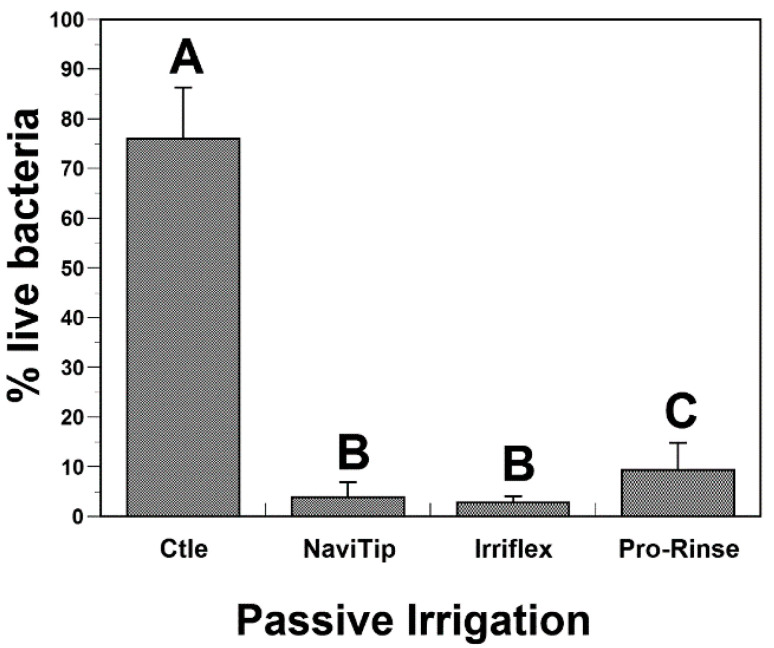
Bacterial viability after passive irrigation using NaviTip, IrriFlex and ProRinse needles. Letters indicate statistical differences among groups (ANOVA, Tukey, 0.05).

A mean percentage of 77% of viable bacteria was reported for untreated control specimens after two weeks in culture. Bacterial viability was significantly reduced after sodium hypochlorite passive irrigation using NaviTip, IrriFlex and ProRinse, compared to controls (Figure 2). During passive irrigation, the use of NaviTip and irriFlex needles allowed for the reduction in bacterial viability below 2% without a statistical difference between these two irrigation needles (*p* > 0.05). The percentage of viable bacteria retrieved from root canal specimens after irrigation with the Pro-Rinse needle was significantly higher (8%) compared to the NaviTip and IrriFlex needles. The hypothesis that the elimination of intracanal *E. faecalis* biofilms would be improved when using a new double-sided vented plastic irrigation needle is therefore rejected.

**Figure 3:**
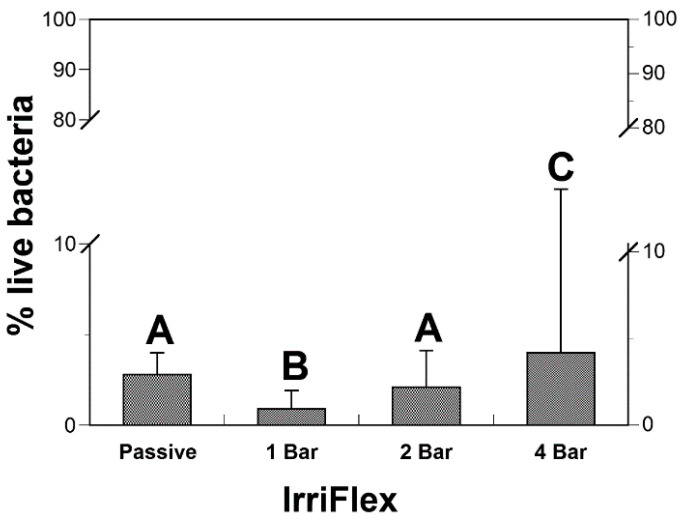
Bacterial viability after passive irrigation using IrriFlex needle or after irrigation under pressure of 1,2 or 4 Bar. Letters indicate statistical differences among groups (ANOVA, Tukey, 0.05).

When the irrigating solution was pressurized at 1 bar (flow rate: 0.13 mL s^−1^), the viability of bacterial biofilms was significantly reduced compared to passive irrigation using the same irrigating needle (*p* < 0.05). Bacterial viability was not statistically different between the passive and 2 bar irrigation groups (*p* > 0.05). At a higher pressure of 4 bar, bacterial viability was significantly higher compared to the other conditions applied during irrigation (Figure 3). The second hypothesis that elimination of intracanal *E. faecalis* biofilms would be improved when higher pressures are used is therefore partially accepted.

## 4. Discussion

Numerous strategies have been used to assess the antibacterial properties of endodontic irrigating solutions, but tests performed on two-week-old mature biofilms were shown to be more pertinent than tests performed over shorter culture periods of time [15,19]. In the current study, a dynamic flow system was developed to allow a long-term culture without excessive bacterial loss and medium evaporation. The elimination of dead bacteria, metabolic end-products and toxic waste throughout the two weeks of culture was made possible. The experimental model used natural teeth rather than artificial substrates such as plastic dishes or hydroxyapatite discs to grow bacterial biofilms [20]. The clinical relevance of the experimental model was further increased by using a closed-end system for root canal irrigation [16].

Despite the fact that nearly all biofilm communities in nature contain multiple microorganisms, *E. faecalis* has been used as a mono-species in our biofilm model. A mono-species biofilm was selected because there is no simple and accurate method to quantify the viability and survival of each bacterial species within a multi-species biofilm due to their competitive interactions over time [21]. Thus, a mono-species biofilm model offered the advantages of simplicity, standardization and control. Undoubtedly, further assessment on multi-species biofilms will ultimately aid with the predicting of clinical performance and safety.

In recent years, flow cytometry (FCM) combined with the fluorescent labeling of bacteria has emerged as a cultivation-independent method for rapid assessment of bacterial viability. Fluorescence readings, indicative of cell membrane integrity, have been used to detect living bacteria inside root canals and dentin tubules and to assess the efficiency of several antibacterial treatments such as sonic powered irrigation or photo-activated disinfection [22,23].

After two weeks in culture, bacterial biofilms remained viable as shown by the high percentage (approx. 75%) of living bacteria (SYTO 9 positive) in control specimens; such stable measurements are in line with previously published data from Tiwari et al. [24] and were expected from the experimental set-up used in this study. Double-stained bacteria (SYTO 9/PI positive), so-called “damaged cells”, were considered as dead bacteria because double-positive bacteria were previously shown to lose their ability to grow on agar plates. [17]. This is particularly relevant considering the risk of selecting resistant E. Faecalis strains that were damaged after exposure to the subinhibitory concentration of antimicrobial agents, as encountered with antibiotics [25].

Concentrations of Sodium hypochlorite ranging between 0.5 and 6% are commonly used for irrigation during root canal preparation. In the current study, we decided to use a 1% sodium hypochlorite solution because there is evidence showing that higher concentrations of chlorine tend to promote the packing of damaged bacteria, which makes the FCM readings more difficult to interpret. As reported by Besmer et al. [26], higher concentrations make the determination of the FCM fingerprint less accurate given the low number of events within the bacterial gate in the density plot. However, it must be pointed-out that a lower chlorine concentration only decreases proteolytic properties but not antibacterial activity. In a recent clinical study, Verma et al. [27] found no difference in healing after endodontic treatment when using 1 or 5% sodium hypochlorite for root canal irrigation. Thus, the aim of this study was not a strict measurement of antibacterial activity per se, but rather a comparison between several irrigation conditions.

The root canals were enlarged to 0.3 mm using ProTaper F3 and sealed apically to better reproduce clinical conditions and simulate a final irrigation. According to Boutsioukis et al. [28], the volume of the root canal, which is estimated to approximately 0.014 mL, can be adequately irrigated using 2 mL of sodium hypochlorite. NaviTip and IrriFlex gave the best results with a slight but not significant advantage for the IrriFlex needle. Undoubtedly, this soft plastic needle had a smoother progression inside the root canals that prevented the wedging of the needle tip as encountered with metal needles. There is evidence showing that a 30 G closed-ended metallic needle is not easily placed within 1 mm from the WL without binding [29]. On the other hand, the ProRinse needle was much less efficient, with approximately a doubled population of bacteria that remained viable after treatment. This could be explained by the design of this needle that allowed the biofilm opposite to the ejection window to better resist the chemo-mechanical action of the irrigating solution. This assumption is somewhat supported by Boutsioukis et al. [5], who showed a reduced laminar flow for the ProRinse irrigation needle compared to open-ended needles in their computational model.

The concept of adding pressure to deliver irrigating solutions at working length and to increase flow rates has been previously described by others. As an example, a negative pressure was used by Nielsen and Baumgartner [30] to suction off the irrigant from the apex towards the crown. In the current study, a positive pressure was applied to the irrigating solution by connecting the irrigation syringe to the pressure device. However, flow rates rather than intra-barrel pressures will be further discussed in this section, because available data on intra-barrel pressure during root canal irrigation is scarce. Also, intra-barrel pressures are subjected to variations particularly during passive manual irrigation using thin 30 G needles. According to Chang et al. [31], the flow rate can vary between 0.08–0.13 mL s^−1^, when using a 28-gauge needle. This favorably compares with a flow rate of 0.06 mL s^−1^ reported for passive irrigation using of narrow 30 G needles and fairly agrees with previously published data by Gopikrishna et al. [32].

The results indicate that not all four (including passive irrigation) flow rate conditions performed equally. When flow rate was set at 0.33 mL s^−1^ (4 Bar condition), a high variability among specimens was reported. It is speculated that an excessive flow rate is deleterious because it reduces the interaction time of the irrigating solution which is rapidly ejected outside of the root canal during injection. Hu et al. [33] have confirmed that slowly moving the needle up and down inside the root canal results in better flush effect and positively influences fluid replacement dynamics.

On the other hand, a flow rate set at 0.13 mL s^−1^ (1 Bar condition) has favored a homogeneous and continuous flow of irrigant that eliminated bacterial biofilm more efficiently. It is therefore suggested to apply a constant pressure to the IrriFlex needle to prevent the collapse of the tip openings of the plastic needle when the syringe tip is packed along the canal walls. This assumption further supports the concept of using the IrriFlex needle during the final irrigation, when shaping is completed, rather than using this needle upon initial canal negotiation.

Needle irrigation allows the main canal to be disinfected, but more complex anatomical areas such as isthmic branches, deltas, and the accessory canal, which are all contaminated niches, would benefit from the use of high flow rates of sodium hypochlorite. Recently, Pereira et al. [34] have shown that an extra high flow irrigation rate resulted in greater biofilm removal than ultrasonic activation devices in the isthmus of an artificial tooth model. Improving the elimination of bacterial biofilms that contaminate root canals during irrigation would undoubtedly improve the success rate of endodontic treatments.

## 5. Conclusions

Within the limits of the current study, it is concluded that NaviTip and IrriFlex were more efficient at removing mature bacterial biofilms than the ProRinse needle, with a slight but not significant advantage for the IrriFlex needle. Adding a constant pressure of 1 bar to the irrigating solution delivered by the IrriFlex needle more efficiently eliminated bacterial biofilm than manual irrigation.
